# Poldip2 knockdown protects against lipopolysaccharide-induced acute lung injury *via* Nox4/Nrf2/NF-κB signaling pathway

**DOI:** 10.3389/fphar.2022.958916

**Published:** 2022-08-31

**Authors:** Yueguo Wang, Wenwen Wang, Shusheng Zhou, Yulan Wang, Obed Cudjoe, Yu Cha, Chunyan Wang, Xiaoguang Cao, Wei Liu, Kui Jin

**Affiliations:** ^1^ Department of Emergency Medicine, First Affiliated Hospital of USTC, Division of Life Sciences and Medicine, University of Science and Technology of China, Hefei, China; ^2^ Graduate School of Bengbu Medical College, Bengbu, China; ^3^ Department of Microbiology and Immunology, School of Medical Sciences, University of Cape Coast, Cape Coast, Ghana; ^4^ Department of Respiratory Disease, Anhui Provincial Chest Hospital, Hefei, China

**Keywords:** Poldip2, acute lung injury, LPS, oxidative stress, inflammation

## Abstract

Polymerase δ-interacting protein 2 (Poldip2) has been reported to mediate acute lung injury (ALI); however, the underlying mechanism is not fully explored. Male C57BL/6 mice and A549 cells were used to establish the lipopolysaccharide (LPS)-induced ALI model, then the expression of Poldip2 and its effect on oxidative stress and the resulting inflammation were detected. Adeno-associated virus serotype 6 (AAV6) mediated Poldip2 knockdown was transfected into mice *via* intratracheal atomization. And A549 cells stimulated with LPS was used to further confirm our hypothesis *in vitro*. ML385, specifically inhibited the activation of the Nrf2 signaling pathway. Our data suggested that LPS stimulation remarkably increased protein levels of Nox4 and p-P65, activities of NADPH and MPO, and generation of ROS, TNF-α, and IL-1β while decreased protein levels of Nrf2 and HO-1 compared with those in NC shRNA + Saline group, which were obviously reversed by Poldip2 knockdown. Concomitantly, Poldip2 knockdown dramatically reduced contents of MDA and enhanced activities of SOD and GSH-Px compared to NC shRNA + LPS group. *In vitro*, we found that knockdown of Poldip2 significantly reversed LPS-induced increase protein levels of Nox4 and p-P65, activity of NADPH, and generation of ROS, TNF-α, and IL-1β, and decrease protein levels of Nrf2 and HO-1, ML385 pretreatment reversed the effects of Poldip2 knockdown mentioned above. Our study indicated that Poldip2 knockdown alleviates LPS-induced ALI *via* inhibiting Nox4/Nrf2/NF-κB signaling pathway.

## Highlights

1) Poldip2 knockdown inhibits LPS-induced ALI in C57BL/6 mice and A549 cells *via* Nox4/ Nrf2/NF-κB signaling pathway.
2) Pretreatment with ML385 reversed the inhibitory effects of Poldip2 knockdown in A549 cells.
3) Poldip2 is an important regulator of oxidative stress and the resulting inflammation and may be a new target for ALI.

## Introduction

Acute lung injury is a life-threatening respiratory disorder in the department of critical care medicine, with a high mortality rate of over 40% (Fan et al., 2018). ALI is characterized by disruption of the alveolar-capillary barrier, impairment of gas exchange, formation of pulmonary edema, and even progressive respiratory failure (Tu et al., 2019). Advances have been made to improve the management of ALI with medical interventions, however, no treatments are currently proved effective for ALI. Therefore, targeting the key protein in inflammatory signaling cascade may represent a promising strategy for ALI.

NADPH oxidase (Nox) family of proteins are considered as a primary enzymatic sources of ROS in respiratory disorders including ALI, which catalyzes the reduction of O2- to ROS and leads to constant oxidative stress (Jarman et al., 2014). NADPH oxidase 4 (Nox4) is the most well-characterized Nox isoform in mammals and is considered to play a fundamental role in lung injury. Prior studies have suggested that inhibition of Nox4 reduced oxidative stress and lung injury caused by various diseases or conditions such as sepsis (Jiang et al., 2020), ischemia-reperfusion (Cui et al., 2018), influenza A virus infection (Yu et al., 2020) and paraquat poisoning (Liu et al., 2019), suggesting Nox4 inhibition exerts protective effects against lung injury.

Poldip2 has attracted a multitude of attention because of its role in a variety of inflammatory conditions, including sepsis-associated encephalopathy (Lv et al., 2017) and ARDS (Fão et al., 2019). Fão and collaborators suggested that heterozygous deletion of the Poldip2 gene significantly attenuates superoxide generation and improves survival in ARDS mouse model, and mechanism analysis indicated that reduced Poldip2 inhibited LPS-induced lung injury *via* regulating mitochondrial ROS-induced inflammatory signaling (Fão et al., 2019). Importantly, a previous study suggested that Poldip2 directly interacted with Nox4 and upregulated Nox4 enzymatic activity and ROS generation (Lyle et al., 2009). However, the mechanism by Poldip2 mediates acute lung injury is poorly understood.

Therefore, we aimed to elaborate the potential mechanism of Poldip2 in LPS-induced ALI, and to underline the relationship between Poldip2 and oxidative stress and the resulting inflammation in mice and A549 cells, consequently to reveal whether Poldip2 knockdown mediates inhibition of Nox4/Nrf2/NF-κB signaling pathway during acute lung injury.

## Materials and methods

### Reagents and chemical

LPS (*Escherichia coli* 055:B5) was provided by Sigma (St. Louis, United States). ML385 was purchased from Med Chem Express (MCE, United States). TNF-α and IL-1β ELISA kits were obtained from Absin (Shanghai, China) and Cusabio (Wuhan, China), respectively. Myeloperoxidase (MPO), malondialdehyde (MDA), superoxide dismutase (SOD) and glutathione peroxidase (GSH-Px) commercial kits were purchased from Jiancheng Corporation (Nanjing, China). NADPH oxidase activity commercial kit was supplied by Genmed Company (Shanghai, China). The ROS detection kit, BCA Protein Assay Kit, and 2′7′-dichloro-dihydro-fluorescein diacetate (DCFH-DA) were provided by the Beyotime Company (Shanghai, China). Antibodies against Poldip2, Nox4, Nrf2, Heme Oxygenase-1 (HO-1), phosphorylated IκB kinase α/β (p-IKKα/β), IKKα, phosphorylated IκBα (p-IκBα), IκB-α, p-P65, and P65 were obtained from Abcam (Cambridge, United States) or Novus (Littleton, United States). Antibody against β-actin was provided by Proteintech Corporation (Wuhan, China).

### Establishment of mice models

**FIGURE 1:**
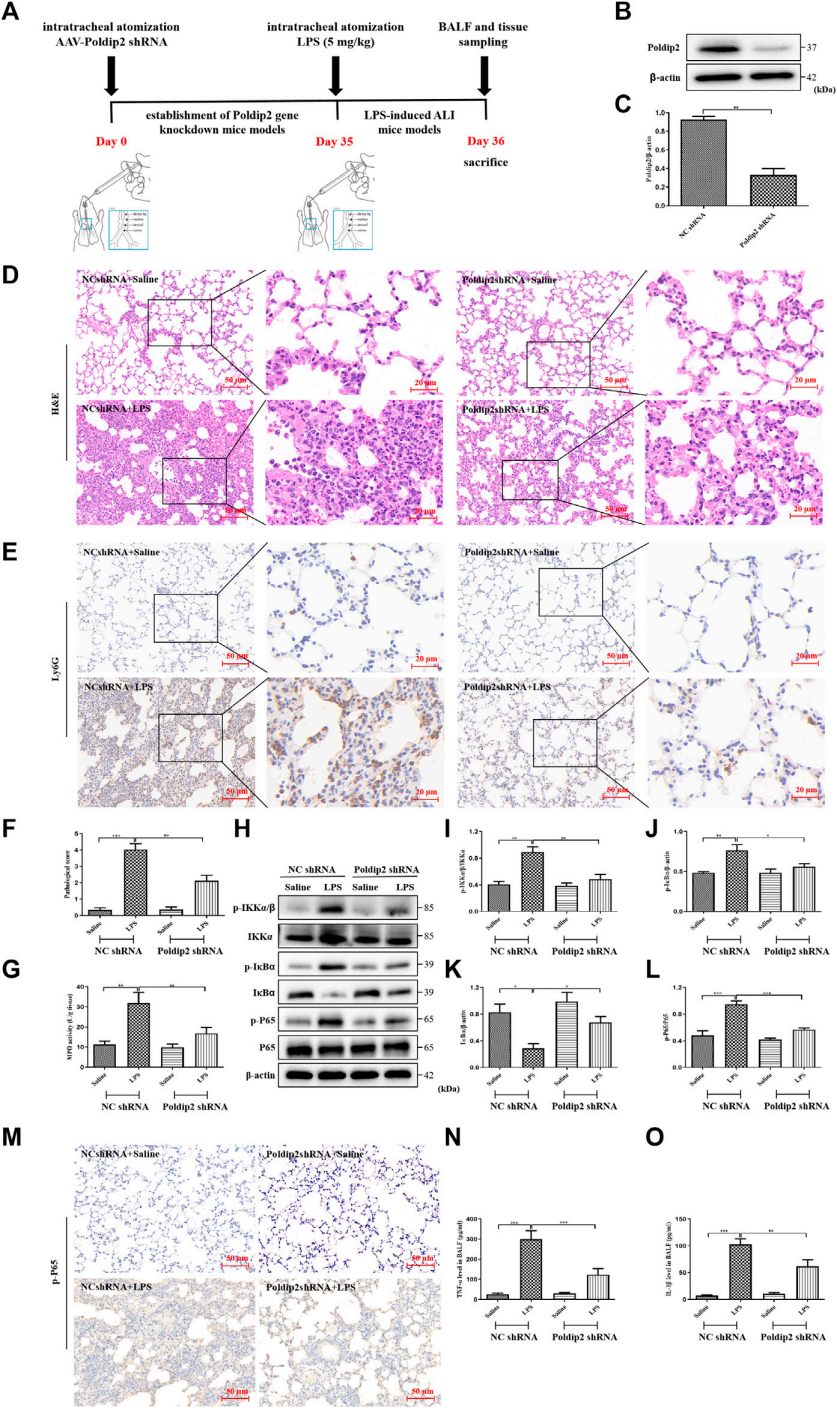
Poldip2 knockdown alleviates inflammation in LPS-induced mice ALI. **(A)** Diagram shows the experimental design. **(B,C)** The protein level of Poldip2 was detected by western blot **(B)** and quantitatively analyzed **(C)**. Male C57BL/6 mice were intratracheally atomized with AAV6 vector expressing Poldip2 shRNA or NC shRNA. Thirty-five days later, 5 mg/kg of LPS or saline was given *via* intratracheal atomization for 24 h **(D)** Representative images showing H&E staining in lung tissue sections, the original image is ×200, the magnified images showed cropped images outlined correspondingly. **(E)** Representative images showing immunohistological staning of Ly6G in lung tissue sections, the original image is ×200, the magnified images showed cropped images outlined correspondingly **(F)** Pathological score of the lungs. **(G)** The MPO activity in lung homogenates were detected. **(H–L)** The protein levels of p-IKKα/β, IKKα, p-IκBα, IκB-α, p-P65, and P65 were detected by western blot **(H)** and quantitatively analyzed **(I–L)**. **(M)** Representative images showing immunohistological staning of p-P65 in lung tissue sections (magnification, ×200). Scale bars are 50 µm. **(N,O)** The levels of TNF-α and IL-1β in BALF were determined by ELISA. *n* = 6 mice per group. Results are represented as mean ± SEM. Statistically significant differences are indicated as ***p < 0.001, **p < 0.01, *p < 0.05.

The experimental protocols were approved by the Ethics of Animal Care and Use of the First Affiliated Hospital of USTC. Male C57BL/6 mice (10–12 weeks old) weighing 20–25 g were randomly assigned to four groups (*n* = 6 per group), then were anesthetized *via* ether inhalation and intratracheally atomized with 6.5 × 1,010 vg/mouse (in 50 µl) of recombinant AAV6 vector expressing Poldip2 short hairpin RNA (shRNA) or NC shRNA (Hanbio, Shanghai, China) following the previously study (Kurosaki et al., 2018). The shRNA sequences of Poldip2 are as follows: Top strand: 5’ -AAT​TCG​CGC​TAT​TGT​ATA​CGC​TTG​GAA​TTC​AAG​AGA​TTC​CAA​G CGT​ATA​CAA​TAG​CGT​TTT​TTG-3′ and Bottom strand: 5’ -GAT​CCA​AAA​AAC​GCT​ATT​GTA TAC​GCT​TGG​AAT​CTC​TTG​AAT​TCC​AAG​CGT​ATA​CAA​TAG​CGC​G-3’. After 35 days, ALI was induced following the previous study (Zhong et al., 2019). Briefly, 5 mg/kg LPS dissolved in 50 µl sterile saline was intratracheally atomized into the lungs of mice. Twenty-4 hours post LPS administation, all mice were sacrificed, then histological and biochemical examinations were carried out (Figure 1A).

### Lung histology and pathological score

The left lungs were lavaged for 3 times with phosphate buffered saline (PBS, 500 μl per time), and bronchoalveolar lavage fluid (BALF) was centrifuged and the supernatants were collected and kept frozen at −80°C, which were used for protein concentrations and inflammatory cytokines detection. Total protein concentrations were detected *via* BCA assay. Part of right lungs were taken and homogenized for western blot assay. The remaining right lungs were excised and fixed in 4% (v/v) paraformaldehyde for 24 h, embedded in paraffin, and sectioned into 5 μm thickness. Histopathological changes were evaluated on lung sections stained with hematoxylin/eosin (H&E).

Pathological scores were performed by two experienced pathology experts blinded to the experimental groups as described previously (Liu et al., 2014; Sun et al., 2021). Five independent random fields (magnification, ×400) were scored per mouse based on the alveolar walls and epithelial thickening, neutrophil infiltration in the alveoli, and elevations in peribronchial and perivascular cuff area. The resulting pathological score is a continuous value between 0 and 5.

### Determination of inflammatory cytokines

The concentrations of TNF-α and IL-1β in BALF supernatants were quantified *via* commercial ELISA kits. In addtion, A549 cells at passages 2–6 were seeded and cultured for 24 h at 50–70% confluence were stimulated with 10 μg/ml LPS for 12 h, the culture medium were harvested for detecting the TNF-α and IL-1β concentration.

### Detection of lung tissues NADPH enzymatic activity

NADPH enzymatic activity was detected according to the manufacturers’ instructions. Briefly, the supernatants of lung homogenates and A549 cells were incubated for 3 min at 30°C with oxidized cytochrome c in a spectrophotometer cuvette, then Nox substrate (NADPH) was mixed together and incubated for 15 min. All values for absorbance was followed at 340 nm by a spectrophotometer.

### Determination of malondialdehyde, superoxide dismutase, glutathione peroxidase and myeloperoxidase

To evaluate the oxidative damage in LPS-induced lung injury, the supernatants of lung homogenate were collected after centrifugation, and the levels of oxidative indicators (MDA and SOD) and antioxidant enzyme (GSH-Px) were determinated using the commercial available kits. Moreover, the activity of MPO was associated with pulmonary neutrophil infiltration and activation, involving in lung inflammation.

### A549 cells culture and RNA interference

The human alveolar epithelial cell line A549 cells were cultured in DMEM with 10% fetal bovine serum (FBS), 100 U/ml penicillin, and 100 mg/ml streptomycin. Cells were maintained at 37°C in a humidified atmosphere with 5% CO2. Lentivirus vectors containing Poldip2-specific shRNA (Poldip2 shRNA) or nonspecific shRNA (NC shRNA) were synthesized by Hanbio Corporation (Shanghai, China). Cells at 30–50% confluence were transducted by lentivirus following the manufacturer’s protocols. The Poldip2 shRNA sequences are listed as follows: Top strand: 5′-GAT​CCG​GCG​CTA​CTG​TAT​CCG​TTT​GGA​GAA​TTC​AAG​AGA​TTC​TCC​AAA​CGG​ATA​CAG​TAG​CGC​CTT​TTT​TG-3′ and Bottom strand: 5′-AAT​TCA​AAA​AAG​GCG​CTA​CTG​TAT​CCG​TTT​GGA​GAA​TCT​CTT​GAA​TTC​TCC​AAA​CGG​ATA​CAG​TAG​CGC​CG-3′.

### Determination of ROS in A549 cells

Production of ROS in A549 cells was detected with oxidation-sensitive fluorimetric probe DCFH-DA as described previously (Chen et al., 2021). Briefly, A549 cells were probed by 10 µM DCFH-DA at 37°C for 20 min, fluorescence emission was determined with CytoFLEX Flow Cytometer (Beckman Coulter, United States). The fluorescent intensities were measured from 10,000 cells at 488 nm (excitation) and 530 nm (emission).

### Immunofluorescence

A549 Cells were fixed with 4% paraformaldehyde for 15 min at room temperature and blocked with 5% bovine serum albumin (BSA) for 2 h at 4°C. Then specific primary antibodies of Nox4 (1: 120), p-P65 (1:400), Nrf2 (1:100) or HO-1 (1: 100) were added and incubated at 4°C. Twelve hours later, appropriate secondary antibodies (1:1000) were added for 1 h, and nuclei were stained with DAPI for 5 min. Photographs were taken using an inverted laser confocal microscope (Carl Zeiss, Germany).

### Immunohistochemistry

The paraffin-embedded sections were routinely deparaffinized and rehydrated. After retrieving antigens with a citrate acid buffer and blocking with 3% H2O2 for 20 min, the sections were incubated with primary antibodies of Nox4 (1:200), Ly6G (1:200), p-P65 (1:200), Nrf2 (1:250) or HO-1 (1:200). Before mounting, the sections were counterstained with hematocylin and subsequently photographed using microscopy (Olympus, Japan).

### Western blot analysis

Protein lysates from lung tissues and A549 cells were extracted and quantified as described previously (Lv et al., 2017). Lysates (30 μg protein) were loaded per lane and separated electrophoretically by 10% SDS-PAGE gels, and transferred onto polyvinylidene fluoride membranes (pre-activated with anhydrous methanol for 1 min), then the membranes were blocked with 5% skim milk in TBST for 1 h and incubated with primary antibodies (1:1,000) for 12 h at 4°C. Appropriate secondary antibodies (1:5000) were added for 1 h at room temperature, immunoreactive bands were visualized with a chemiluminescence detection kit.

### Statistical analysis

Data were analysed by Graph Pad Prism 8.0 software. Data was presented as mean ± SEM of three independent experiments. Student’s *t*-test was conducted to evaluate differences between two groups, and ANOVA was used to for multiple groups. *p* < 0.05 was considered statistically significant.

## Result

### Poldip2 knockdown alleviates lipopolysaccharide-induced lung inflammation in acute lung injury mice

We first determined the expression of Poldip2 in Poldip2 gene knockdown mice. Recombinant AAV6 vector expressing Poldip2 shRNA or NC shRNA was administered *via* intratracheal atomization in mice. Thirty-five days after administration of AAV6-mediated shRNA, western blot analysis showed that Poldip2 knockdown mice showed a remarkable decrease in Poldip2 expression compared to the NC shRNA group (Figures 1A–C).

To confirm whether Poldip2 knockdown alleviates lung inflammation in mice, NC shRNA and Poldip2 shRNA mice were challenged with saline or LPS for 24 h, then histopathologic examination was performed (Figure 1D). We next evaluated the pathological score during lung injury in mice and found that Poldip2 knockdown markedly decreased the pathological score compared with those in NC shRNA + LPS group (Figure 1F). In addition, neutrophil (Ly6G) infiltration was examined *via* immunohistochemical assay (McCracken et al., 2020). Our results showed a significant increase of neutrophil inflitration in alveolar and interstitial spaces in NC shRNA + LPS group as compared with those in NC shRNA + Saline group, whereas this histopathologic feature was attenuated in Poldip2 knockdown mice (Figure 1E). The MPO activity was detected in lung tissues to further confirm the histopathological findings. The results suggested that activity of MPO was significant decreased in Poldip2 shRNA + LPS group compared with those in NC shRNA + Saline group (Figure 1G).

The nuclear factor kappa B (NF-κB) pathway is an prototypical proinflammatory signaling pathway that regulates inflammatory cytokines production under LPS challenge (Ling et al., 2020). Therefore, we next investigated whether knockdown of Poldip2 reduced lung inflammation *via* downregulating the IKK/IκB/NF-κB signaling pathway. Western blot showed Poldip2 knockdown significantly reversed LPS stimulation induced increases in protein levels of the p-IKKα/β, p-IκBα, and p-P65 and decreases in protein level of the IκBα (Figures 1H–L). Moreover, similar results were found in our immunohistochemical assays (Figure 1M). Additionally, BALF samples were collected to determine the levels of inflammatory cytokines in mice. As shown in Figures 1N,O, we observed that Poldip2 knockdown remarkably inhibited LPS-induced increases in TNF-α and IL-1β levels in BALF in ALI mice. Collectively, our results suggested that Poldip2 knockdown attenuated LPS-induced lung inflammation *via* inhibits NF-κB signaling pathway.

### Poldip2 knockdown ameliorates lipopolysaccharide-induced oxidative stress in acute lung injury mice

**FIGURE 2:**
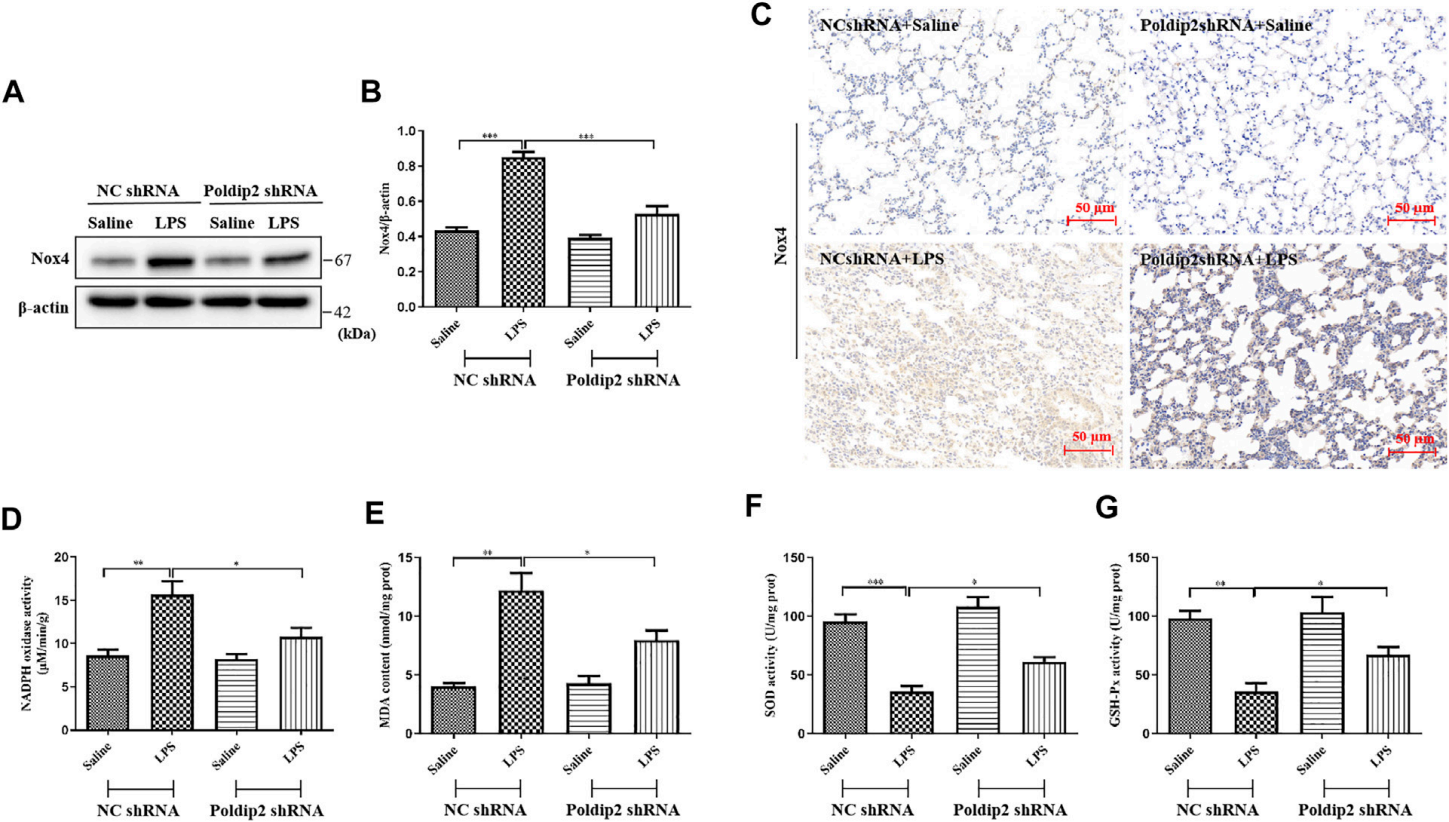
Poldip2 knockdown suppresses oxidative stress in LPS-induced mice ALI. **(A)** Male C57BL/6 mice were intratracheally atomized with AAV6 vector expressing Poldip2 shRNA or NC shRNA. Thirty-five days later, 5 mg/kg of LPS or saline was given *via* intratracheal atomization for 24 h **(A,B)** The protein level of Nox4 were detected by western blot **(A)** and quantitatively analyzed **(B) (C)** Representative images showing immunohistological staning of Nox4 in lung tissue sections (magnification, ×200). Scale bars are 50 µm **(D)** The NADPH oxidase activity in lung homogenates was detected by spectrophotometer. **(E–G)** The MDA concentrations and SOD and GSH-Px activities in lung homogenates were determined. *n* = 6 mice per group. Results are represented as mean ± SEM. Statistically significant differences are indicated as ***p < 0.001,**p < 0.01, *p < 0.05.

Nox4 is a major source of ROS and has been reported to be rapidly activated under LPS challenge (Fisher et al., 2019; Jiang et al., 2020). Herein, we aimed to elucidate the mechanisms that underlie Poldip2 regulates protein level and oxidase activity of Nox4. The results indicated that Nox4 protein level and NADPH oxidase activity were markedly attenuated in Poldip2 shRNA group compared with those in NC shRNA group following LPS challenge (Figures 2A,B,D), indicating that Poldip2 upregulates Nox4 gene. Similar results were found in our immunohistochemical assays (Figure 2C).

Furthermore, to determine whether Poldip2 knockdown attenuates LPS-induced oxidative stress, levels of MDA, SOD, and GSH-Px, as indicators of lipid peroxidation and the activity of antioxidant enzymes, were detected in lung tissues, respectively. As shown in Figures 2E–G, in response to LPS stimulation, the content of MDA was absolutely increased, while the activities of SOD and GSH-Px were remarkably reduced compared with those in NC shRNA + Saline group, but were reversed in Poldip2 shRNA + LPS group. Collectively, our data showed that Poldip2 knockdown suppresses oxidative stress in ALI mice.

### Poldip2 knockdown attenuates lipopolysaccharide-induced acute lung injury through Nrf2 Signaling pathway activation

**FIGURE 3:**
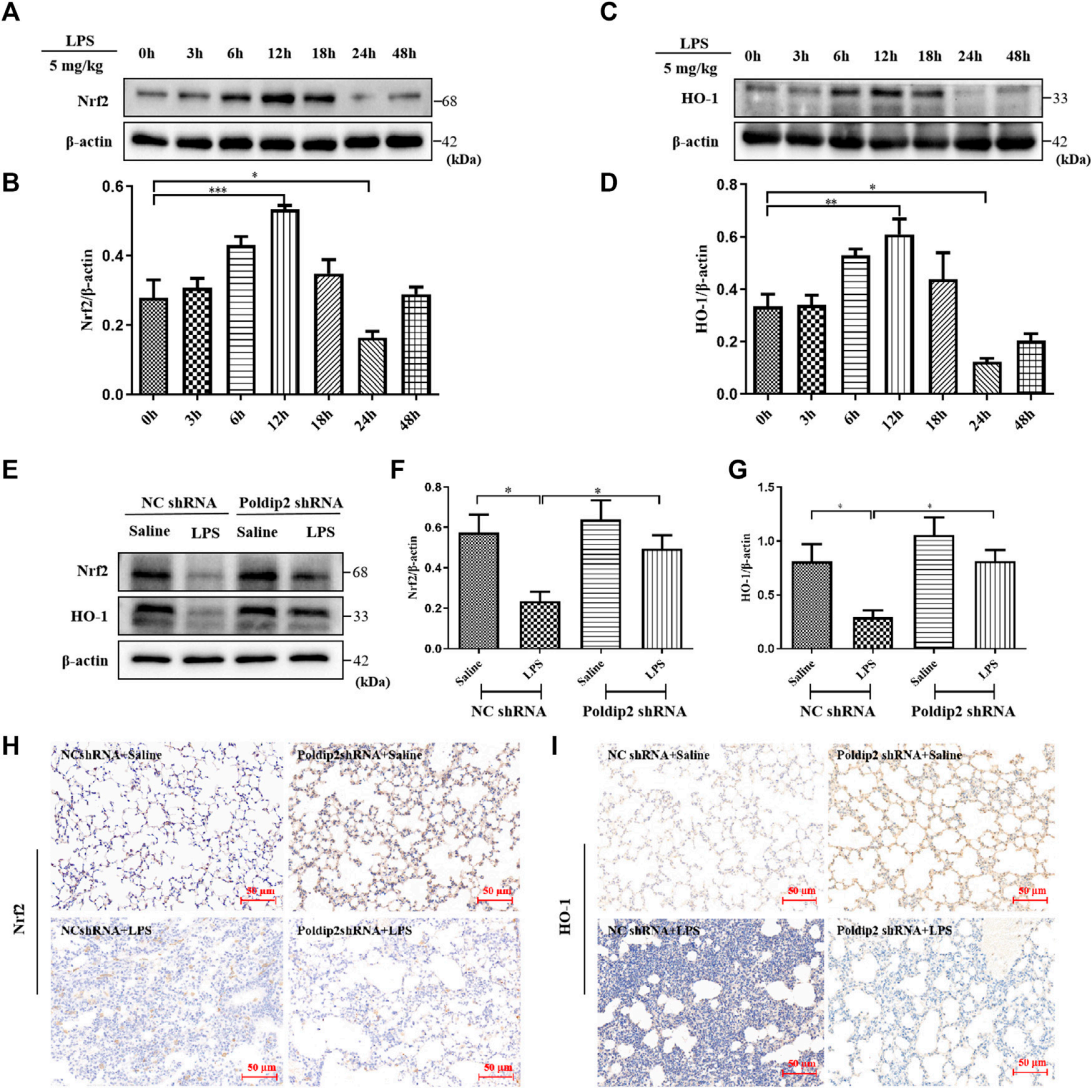
Poldip2 knockdown inhibits LPS-induced ALI through activating Nrf2 signaling pathway. Male C57BL/6 mice were intratracheally atomized with 5 mg/kg of LPS for different times (0–48 h) **(A–D)**. The protein levels of Nrf2 and HO-1 were detected by western blot **(A,C)** and quantitatively analyzed **(B,D)**. Male C57BL/6 mice were intratracheally atomized with AAV6 vector expressing Poldip2 shRNA or NC shRNA. Thirty-five days later, 5 mg/kg of LPS or saline was given *via* intratracheal atomization for 24 h **(E–G)** The protein levels of Nrf2 and HO-1 were detected by western blot **(E)** and quantitatively analyzed **(F,G) (H,I)** Representative images showing immunohistological staning of Nrf2 and HO-1 (magnification, ×200). Scale bars are 50 μm *n* = 6 mice per group. Results are represented as mean ± SEM. Statistically significant differences are indicated as ***p < 0.001,**p < 0.01, *p < 0.05.

Mounting evidence has indicated that Nrf2/HO-1 signaling pathway is an important endogenous antioxidant defense system and participates in regulation of oxidative stress and inflammation (Ahmad et al., 2020; Yao et al., 2020). Herein, we evaluated the effect of LPS stimulation on protein levels of Nrf2 and HO-1 in lung tissues. Mice were treated with LPS at different times (0–48 h). As shown in Figures 3A–D, we observed an increase in Nrf2 and HO-1 above baseline at 3 h, peaked at 12 h and decreased below baseline at 24 h. Our results indicated that LPS challenge induced Nrf2 and HO-1 expression in a time-dependent manner in mice.

Additionally, the effects of Poldip2 knockdown on the Nrf2/HO-1 pathway were detected *via* western blot and immunohistochemical assays. As shown in Figures 3E–G, LPS challenge significantly inhibited protein levels of Nrf2 and HO-1 compared with those in NC shRNA + Saline group, which were reversed in Poldip2 shRNA + LPS group. Results obtained from immunohistochemistry analysis (Figures 3H,I) were consistent with Western blot experiments (Figures 3E–G). Collectively, these results indicated that Poldip2 knockdown could reverse LPS-induced Nrf2 signaling pathway.

### Poldip2 knockdown inhibits lipopolysaccharide-induced oxidative stress and inflammation in a549 cells

**FIGURE 4:**
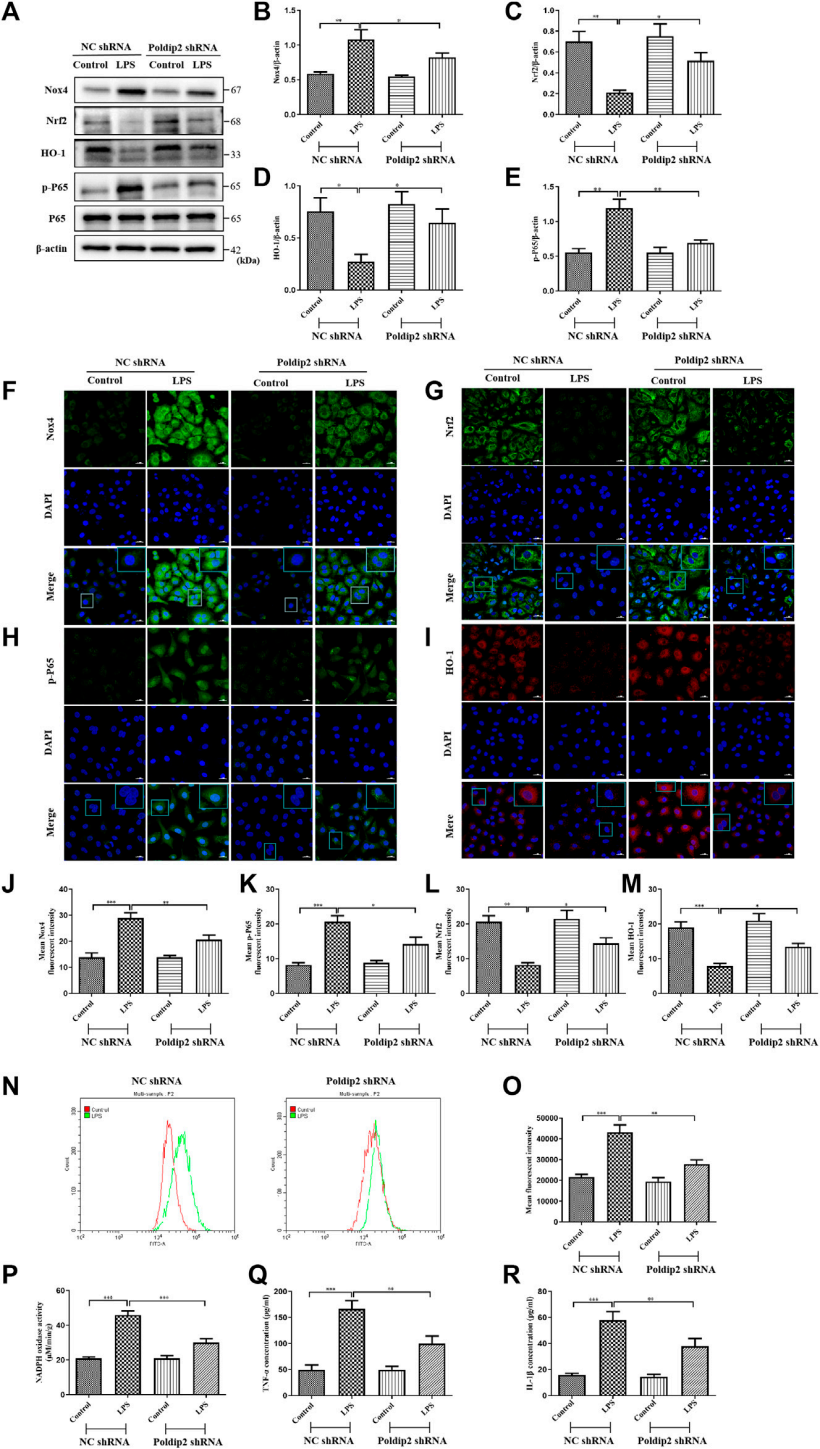
Poldip2 knockdown inhibits LPS-induced oxidative stress and the resulting inflammation in A549 cells. Confluent A549 cells were transfected with lentivirus vector expressing NC shRNA or Poldip2 shRNA for 72 h, and treated with PBS or 10 μg/ml LPS for 12 h. **(A–E)** The protein levels of Nox4, Nrf2, HO-1, p-P65 and P65 were detected by western blot **(A)** and quantitatively analyzed **(B–E)**. **(F–M)** Representative images showing immunofluorescence staning of Nox4, p-P65, Nrf2 and HO-1. Cells were stained with Nox4, p-P65 or Nrf2 (green) and HO-1(red), and counter-stained with DAPI (blue) (magnification of ×400), Scale bars are 20 µm. **(N,O)** The generation of intracellular ROS were detected by flow cytometry. **(P)** The NADPH oxidase activity in A549 cells was detected by spectrophotometer. **(Q,R)** The levels of TNF-α and IL-1β in the supernatant were detected by ELISA. Results are represented as mean±SEM of 3 independent experiments. Statistically significant differences are indicated as ***p < 0.001, **p < 0.01, *p < 0.05.

To further determine whether Poldip2 mediates oxidative stress and inflammation is *via* regulating the Nox4/Nrf2/NF-κB signaling pathway. A549 cells were transfected with either NC shRNA or Poldip2 shRNA and stimulated with PBS or 10 μg/ml LPS for 12 h. Our study suggested that increased protein levels of Nox4 and p-P65, as well as decreased protein levels of Nrf2 and HO-1 were detected under LPS challenge compared with those in NC shRNA group. Conversely, knockdown of Poldip2 notably reduced Nox4 and p-P65 protein levels and increased Nrf2 and HO-1 protein levels compared with those in NC shRNA + LPS group (Figures 4A–E). Similar results were obtained in our immunofluorescence assays, suggesting that knockdown of Poldip2 attenuates Nox4 and p-P65 expression (Figures 4F,H,J,K), but enhanced Nrf2 and HO-1 expression (Figures 4G,I,L,M) in response to LPS stimulation.

Then, we evaluated the role of Poldip2 knockdown on ROS generation, NADPH oxidase activity, and TNF-α and IL-1β generation in LPS-treated A549 cells. After LPS stimulation for 12 h, cells were probed by 10µM DCFH-DA at 37°C for 20 min, and ROS generation was determined with flow cytometry. We found that LPS stimulation substantially induced ROS generation compared to those in NC shRNA group, but was inhibited by Poldip2 knockdown (Figures 4N,O). Furthermore, knockdown of Poldip2 significantly suppressed LPS-induced increase of NADPH oxidase activity and generation of TNF-α and IL-1β compared with those in NC shRNA + LPS group (Figures 4P–R). Taken together, the present results suggested that Poldip2 knockdown suppresses LPS-induced oxidative stress and inflammation in A549 cells.

### The Nrf2 inhibitor ML385 attenuates the inhibitory action of Poldip2 knockdown in lipopolysaccharide-stimulated A549 cells

**FIGURE 5:**
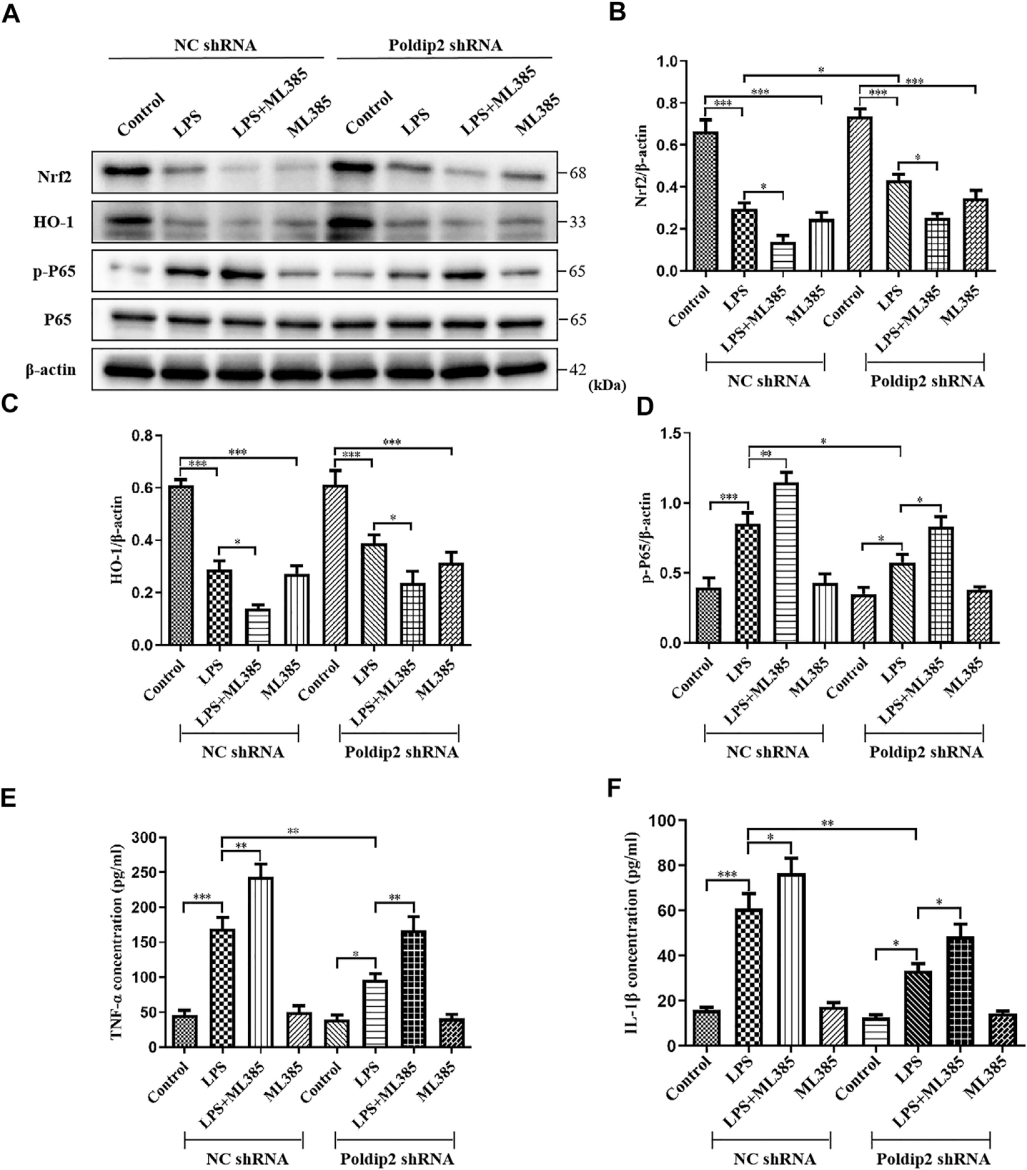
Protective effects of Poldip2 knockdown in attenuating oxidative stress and the resulting inflammation are reversed by ML385. Transfected A549 cells (NC shRNA or Poldip2 shRNA) were pretreated with or without 5 μM ML385 for 1 h, and treated with 10 μg/ml LPS for 12 h **(A–D)** The protein levels of Nrf2, HO-1, p-P65 and P65 were detected by western blot **(A)** and quantitatively analyzed **(B–D)**. **(E,F)** The levels of TNF-α and IL-1β in the supernatant were detected by ELISA. Results are represented as mean ± SEM of three independent experiments. Statistically significant differences are indicated as ***p < 0.001,**p < 0.01, *p < 0.05.

To further determine whether Poldip2 knockdown inhibited LPS-induced NF-κB signaling pathway activation was dependent on Nrf2 protein, Nrf2 signaling inhibitor ML385 was used *in vitro*. Transfected A549 cells (NC shRNA or Poldip2 shRNA) were pretreated with or without 5 μM ML385 for 1 h then incubation with 10 μg/ml LPS for 12 h. As shown in Figures 5A–C, LPS challenge dramatically decreased protein levels of Nrf2 and HO-1 as compared to those in NC shRNA or Poldip2 shRNA group, while administration of ML385 further enhanced these effects, suggesting ML385 pretreatment could block Nrf2 signaling pathway. In addition to that, the protein level of p-P65 significant increased under LPS challenge were further upregulated by ML385 pretreatment (Figures 5A,D), suggesting that Nrf2 acted as an upstream regulator of p-P65 and its activation contributed to inhibit NF-κB signaling pathway.

Then, we evaluated whether LPS-induced increase of inflammatory cytokines generation were further enhanced by ML385 pretreatment in A549 cells. As shown in Figures 5E,F, knockdown of Poldip2 remarkably suppressed generation of TNF-α and IL-1β compared with those in NC shRNA + LPS group. Importantly, compared to LPS-challenged groups, pretreatment with ML385 further increased generation of TNF-α and IL-1β. Collectively, these findings suggested that ML385 attenuated the inhibition of Poldip2 knockdown in LPS-induced NF-κB signaling pathway activation in A549 cells.

## Discussion

In this study, we showed the beneficial effects of Poldip2 knockdown against LPS-induced oxidative stress and the resulting inflammation in mice and A549 cells *via* inhibiting of Nox4/Nrf2/NF-κB signaling pathway. Pretreatment with ML385 significantly reversed the inhibitory effects of Poldip2 knockdown in A549 cells.

It has been established that Poldip2 is emerging as a novel therapeutic target for several inflammatory disorders (Forrester et al., 2019; Kikuchi et al., 2019). Recent evidence indicated that Poldip2 deficiency attenuated pulmonary edema and vascular inflammation in a LPS-induced ARDS mouse model *via* inhibiting mitochondrial ROS production (Forrester et al., 2019). Consistent with this data, our results suggested that LPS-induced neutrophil infiltration and histopathological damage were markedly alleviated in Poldip2 knockdown mice. Furthermore, it is widely known that the recruitment of neutrophil generates MPO, which is considered to be major pathogenic components in ALI (Chniguir et al., 2019). Here, our data indicated that knockdown of Poldip2 obviously decreased LPS-induced MPO production.

Invariably, NF-κB activition is a crucial regulator of the stress and immune responses, proliferation, inflammation, differentiation, and apoptosis (Oeckinghaus et al., 2011; Li et al., 2021). A previous study has indicated that phosphorylation of NF-κB P65 leaded to the generation of TNF-α and IL-1β, and NF-κB pathway inhibition was proven to alleviate ALI (Huang et al., 2018). Herein, we found that knockdown of Poldip2 effectively reduced protein levels of p-IKKα/β, p-IκBα, and p-P65 but enhanced protein level of IκBα in ALI mice, suggesting that the protective effects of Poldip2 knockdown in alleviating LPS-induced inflammation may be in relation to NF-κB signaling inactivation. In addition, the generation of cytokines in BALF is one of the hallmarks during ALI. Our data also suggested that levels of TNF-α and IL-1β in BALF were substantially inhibited in Poldip2 knockdown mice compared with those in NC shRNA + LPS mice. Taken together, the obtained results demonstrated that the contribution of Poldip2 knockdown to LPS-induced lung inflammation was at least associated with the downregulation of neutrophil infiltration and cytokines production.

Numerous studies have implicated oxidative stress exerts an important role in the development of ALI (Gao et al., 2020; Meng et al., 2021). Nox4-derived ROS leads to sustained oxidative stress may be one important mechanism involved (Cui et al., 2018; Jiang et al., 2020; Yu et al., 2020). Poldip2, a newly discovered Nox4 enhancer protein, had attracted widespread attention (Lyle et al., 2009). Our previous study showed that Poldip2 interacted with Nox4 and mediated oxidative stress and inflammation in LPS-stimulated A549 and Beas-2B cells (Wang et al., 2022), indicating a plausible underlying mechanism of excessive oxidative stress leading to cellular injury and that the molecular events are associated with Poldip2. Collectively, we found that Poldip2 knockdown attenuated the activation of LPS on Nox4 protein level and NADPH oxidase activity in ALI mice.

In addition, excessive oxidative stress reflects an oxidant and antioxidant imbalance in the organism (Sies, 2015). MDA, a product of polyunsaturated fatty acids peroxidation, is commonly known as an marker in evaluating the degree of oxidative stress (Tsikas, 2017). While the antioxidant enzymes (SOD and GSH-Px) are known to eliminate the toxic effects of superoxide radicals, their activities is related to oxidative stress (SreeHarsha, 2020). Herein, we observed that increased MDA content and reduced SOD and GSH-Px activities in ALI mice were reversed by Poldip2 knockdown, suggesting that knockdown of Poldip2 protects mice against accelerated LPS-induced oxidative stress *via* enhancing antioxidant enzymes expression and inhibiting lipid peroxidation.

Nuclear factor erythroid 2-related factor 2(Nrf2), a redox-sensitive transcription factor involves in antioxidative and cytoprotective pathways and is a primary target of many diseases (Fão et al., 2019; Liu et al., 2019; He et al., 2020). Mounting studies indicated that Nrf2 pathway activation effectively protected the lung tissues from oxidative stress and inflammation under LPS stimulation (Lv et al., 2017; Huang et al., 2020). As oxidative stress occurs, the Nrf2 activation contributes to induction of antioxidant enzymes (SOD and GSH-Px) and its downstream antioxidant protein (HO-1), which eventually exerts antioxidant and protective functions under ALI. Previously studies demonstrated that Nox restrained gastric aspiration-induced ALI *via* a Nrf2-dependent mechanism (Davidson et al., 2013). In this study, our study suggested that Nrf2 signaling pathway was activated under LPS challenge in a time-dependent manner, it decreased below the baseline at 24 h after LPS stimulation, similar result has been found in HO-1 protein expression. Excessive production of ROS under pathological conditions leaded to the exhaustion of Nrf2 and reduced the expression of HO-1 confirmed our observations, similar results have been observed in LPS-induced ALI mice (Fei et al., 2019; Huang et al., 2019; Yang et al., 2019). More importantly, we observed that knockdown of Poldip2 effectively reversed LPS-induced inhibition of Nrf2 and HO-1, suggesting that the protective effects exhibited by Poldip2 knockdown against oxidative stress and the resulting inflammation under LPS stimulation may associated with the Nrf2 signaling pathway activation.

Furthermore, ALI is the clinical syndrome associated with histopathological diffuse alveolar damage, and oxidative stress can exaggerate disruption of alveolar cells (Gong et al., 2018). Based on the above observations, our data further investigated the underlying molecular mechanisms of Poldip2 knockdown on oxidative stress and the resulting inflammation under LPS stimulation *in vitro*. Herein, we constructed lentiviral vectors expressing Poldip2 shRNA or NC shRNA and transfected into A549 cells and incubated with LPS for 12 h. Consistent with our *in vivo* studies, the results indicated that Poldip2 knockdown notably inhibited Nox4/Nrf2/NF-κB signaling pathway in A549 cells under LPS-stimulation. Importantly, we also revealed that knockdown of Poldip2 absolutely attenuated LPS-induced ROS production, and TNF-α and IL-1β activation. In addition, we further explored whether Poldip2 knockdown mediated inhibition of inflammatory cytokines was dependent on the Nrf2 signaling pathway activation. ML385, a small molecular Nrf2 inhibitor, which directly interacts with Nrf2 protein and blocks its downstream genes expression has been used (Qiu et al., 2020). Our results suggested that ML385 pretreatment further enhanced LPS-induced NF-κB pathway activation and cytokines generation in A549 cells transfected with Poldip2 shRNA. In line with our findings, previously studies suggested that Nrf2 deficiency induced an exacerbation of inflammation including sepsis and intestinal ischemia/reperfusion induced ALI (Thimmulappa et al., 2006; Yan et al., 2018).

## Conclusion

In summary, our present study suggests that Poldip2 knockdown is required to inhibit Nox4/Nrf2/NF-κB signaling pathway in LPS-induced ALI in mice and A549 cells. Moreover, Poldip2 is crucial for regulating oxidative stress and the resulting inflammation, and may be a new target for ALI.

## Data Availability

The original contributions presented in the study are included in the article/supplementary material, further inquiries can be directed to the corresponding author.
